# Raman and fourier transform infrared spectroscopy techniques for detection of coronavirus (COVID-19): a mini review

**DOI:** 10.3389/fchem.2023.1193030

**Published:** 2023-05-18

**Authors:** Qiuqi Zhang, Lei Zhao, Guoliang Qi, Xiaoru Zhang, Cheng Tian

**Affiliations:** ^1^ The First School of Clinical Medicine, Southern Medical University, Guangzhou, China; ^2^ Shandong Provincial Key Laboratory of Detection Technology for Tumor Markers, Collaborative Innovation Center of Tumor Marker Detection Technology, Equipment and Diagnosis-Therapy Integration in Universities of Shandong, College of Chemistry and Chemical Engineering, Linyi University, Linyi, China; ^3^ Key Laboratory of Optic-Electric Sensing and Analytical Chemistry for Life Science, MOE, Shandong Key Laboratory of Biochemical Analysis and College of Chemistry and Molecular Engineering, Qingdao University of Science and Technology, Qingdao, China

**Keywords:** COVID-19, diagnostics, Raman spectroscopy, FTIR spectroscopy, SERS, ATR-FTIR

## Abstract

Coronavirus pandemic has been a huge jeopardy to human health in various systems since it outbroke, early detection and prevention of further escalation has become a priority. The current popular approach is to collect samples using the nasopharyngeal swab method and then test for RNA using the real-time polymerase chain reaction, which suffers from false-positive results and a longer diagnostic time scale. Alternatively, various optical techniques, namely, optical sensing, spectroscopy, and imaging shows a great promise in virus detection. In this mini review, we briefly summarize the development progress of vibrational spectroscopy techniques and its applications in the detection of SARS-CoV family. Vibrational spectroscopy techniques such as Raman spectroscopy and infrared spectroscopy received increasing appreciation in bio-analysis for their speediness, accuracy and cost-effectiveness in detection of SARS-CoV. Further, an account of emerging photonics technologies of SARS-CoV-2 detection and future possibilities is also explained. The progress in the field of vibrational spectroscopy techniques for virus detection unambiguously show a great promise in the development of rapid photonics-based devices for COVID-19 detection.

## Introduction

Since the winter of 2019, the epidemic of severe acute respiratory syndrome coronavirus 2 (SARS-CoV-2) has turned into a hot topic in the whole society. It was first observed at Wuhan City, Hubei Province, on 31 December 2019 and has spread worldwide ever since (WHO Coronavirus, 2022). SARS-CoV-2 tends to outbreak in people-intensive public places like school and hospital (Oliver, 2021; Pagel, 2021), as the transmission is commonly occurred through pathogen-carried respiratory droplets and contact with contaminated object (Wiersinga et al., 2020). Moreover, the phenomenon of that some cases happen between medical team indicate that infection via aerosol exhaled by patient is popular as well (Hamilton, 2021). Fever, dry cough, shortness of breath and fatigue are the most common clinical manifestations (Pereira et al., 2021). SARS-CoV-2 also lead to vascular dysfunction that may result in erectile dysfunction in men; loss of grey matter and shrinkage of hippocampus was detected among elders. Thus, an accurate and rapid detection method is crucial under such a scale of pandemic.

At present, Real-time polymerase chain reaction (RT-PCR) is considered as the gold standard for SARS-CoV-2 detection due to its outstanding sensitivity (Jadhav et al., 2021; Qiblawey et al., 2021). Real-time detection based primarily on detecting the presence of viral RNA with a fluorescence spectrophotometer during PCR (Corman et al., 2020). However, it suffers from defects such as being time-intensive, highly-demanding transportation process and complex procedure of sample treatment (Yakoh et al., 2021). As the coronavirus 2019 (COVID-19) is airborne, complex sample handling procedures can bring about greater risk of transmission. Moreover, the precision rate of RT-PCR is not 100% since errors may occur due to virus mutation or sample contamination (Kashefi-Kheyrabadi et al., 2022). Therefore, there is an urgent need to improve the speed, cost effectiveness and accuracy of detecting coronavirus infections thus inhibiting the spread of the disease.

For the purpose of non-invasive diagnostic applications, vibrational spectroscopy techniques have drawn new applications in biotechnology, science and medicine research (Pence and Mahadevan-Jansen, 2016; Baker et al., 2018). In this mini-review, we focus on the vibrational spectroscopy techniques which obtain the chemical structure of molecules by observing the molecular vibrations. From immune system response, the vibrational profiles of viral or antibody proteins can be obtained (Leal et al., 2018). Among the vibrational spectroscopy techniques, there has been growing interest in applying Raman spectroscopy (RS) and infrared spectroscopy (IR) for detecting and monitoring infections (Movasaghi et al., 2008; Talari et al., 2015; Harrison and Berry, 2017). Here, we are going to discuss the two approaches above for efficient detection of COVID-19. We hope that this mini-review will demonstrate the broad promise of RS and IR in detecting viruses that cause respiratory disease and help researchers build advanced diagnostic systems to overcome pandemics.

### Raman spectroscopy sensors

**FIGURE 1:**
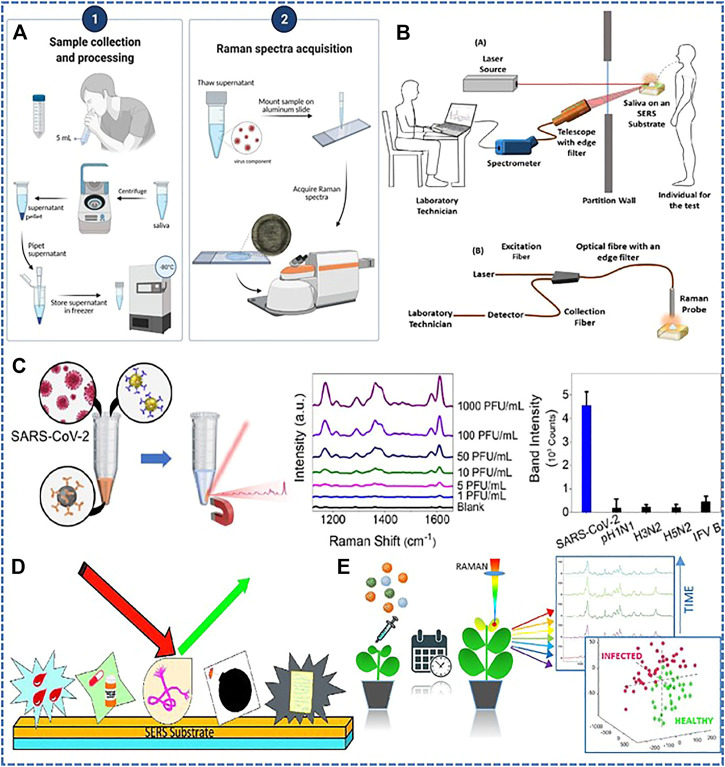
Application of surface-enhanced Raman technique in virus detection. **(A)** Workflow from saliva collection to determination of COVID infection status from Raman spectra. Copyright^©^ 2022, International Society for Optical Engineering. **(B)** The use of Raman/laser-induced fluorescence spectroscopy devices has the potential to detect viruses through saliva at a safer distance. Copyright^©^ 2021, Elsevier. **(C)** A schematic of the application of SERS active substrates to detect biological and chemical materials. Copyright^©^ 2018, Elsevier. **(D)** A platform based on Raman spectroscopy combined with chemometric analysis to monitor tomato infection by two dangerous and different viral pathogens, namely, tomato yellow leaf curl Sardinia virus (TYLCSV) and tomato spotted wilt virus (TSWV). Copyright^©^ 2022, Elsevier. **(E)** Schematic illustration of the magnetically assisted SERS immunoassay for SARS-CoV-2. Copyright^©^ 2019 American Chemical Society.

Raman spectroscopy has received a lot of attention in biological study. Raman scattering is the inelastic scattering of photons from vibrating molecules. After interacting with molecular bond, the frequency of the photon changes. This difference in frequency provides information about the molecular vibrations (Liu et al., 2022; Wang et al., 2022). Raman spectroscopy has been used to detect RNA viruses present in serum samples like hepatitis B virus (HBV) (Khan et al., 2018; Tong et al., 2019). It also has potential application in detecting COVID-19 since this spectroscopy can give chemical fingerprints of molecules. The virus existed in human biofluids which carry biomarkers when people got infection. These biomarkers like carbohydrates, lipids, hormones, nucleic acids can be identified with Raman spectroscopy (Zhang et al., 2005). (Yin et al., 2021) verified that it could be applied for a safe and efficient method for COVID-19 detection by analyzing serum specimens of confirmed patients, suspected patients and healthy individual on Raman spectroscopy. Moreover, it showed a relatively high accuracy in identifying COVID-19 symptomatic and asymptomatic individuals, which indicated that Raman spectroscopy could be a promising tool for COVID-19 screening. (Goulart et al., 2022). also analyzed serum samples for identification of COVID-19 by means of Raman spectroscopy. The study focused on the fact that the COVID-19 serum samples had an increase in blood lipids, nitrogen compounds and nucleic acids, while content of protein and tryptophan decreased, which also illustrated Raman spectroscopy as a rapid and economic detection platform. Since hematological disease is a common symptom of COVID-19 (Bergamaschi et al., 2021), (Revin et al., 2022) combined Raman spectroscopy and laser interference microscopy techniques to specifically identify decreased erythrocyte oxygen transport function and abnormal appearance in patients with confirmed COVID-19 compared with healthy people. In addition to blood tests, other body fluids like saliva can also get more accurate results. (Carlomagno et al., 2021). and (Ember et al., 2022) focused on analyzing saliva samples and had the potential to significantly distinguish the patients currently infected with COVID-19 from healthy subjects and/or previously infected subjects. As shown in Figure 1A, saliva samples were centrifuged and the supematant was stored at −80°C for clarity, then it was thawed and mounted on an aluminum slide to acquire spectra via a Raman spectrometer. Collecting saliva specimens as observed biofluids has a number of advantages over serum testing, especially for its minimal invasiveness and no pain involved. In addition, (Lukose et al., 2021), reported using a Raman spectroscopy device to detect the virus by saliva at a safer distance, which may be able to work as an isolation spectroscopy technique and address the major problem of virus transmission among health sampling workers during sample collection in places with high density people (Figure 1B). Other biofluids like urine also attracted researchers’ interest, (Robertson et al., 2022), realized detection of complex multi-molecular fingerprints in urine samples associated with COVID-19 disease and reached an overall prediction accuracy of 97.6%. (Jadhav et al., 2021). proposed a diagnostic scheme based on surface enhanced Raman spectroscopy (SERS) and a microfluidic device containing integrated microchannels functionalized by vertically aligned gold/silver-coated carbon nanotubes. As seen in Figures 1C,D, Surface Enhanced Raman Scattering (SERS) is a molecular fingerprint spectrum analysis technique that can generate molecular fingerprint spectra with a great sensitivity of single molecule detection, thus being widely applied in identifying biological molecule, respiratory virus, animal virus (Fikiet et al., 2018; Mandrile et al., 2019; Eskandari et al., 2022; Gao et al., 2023). As shown in Figure 1E, gold and silver nanoparticles were usually used as active substrates to obtain high sensitivity (Cha et al., 2022). This scheme was expected to successfully capture viruses from a variety of biofluids, such as saliva, naso discharge, tears and others. Notably, a technique mainly utilizing the phenomenon of SERS was proposed by (Pramanik et al., 2021b). The authors described the development of gold nanoparticles with anti-spike antibodies for rapid diagnosis of COVID-19 viruses within 5 min, which allowed the naked eye to observe the presence of virus. Moreover, the antigen–antibody interaction prevented the virus from integrating with cell membranes thus restraining virus transmission. Similarly, (Yang et al., 2021), proposed a “virus-traps” nanostructure consisting of oblique gold nanoneedles modified with Human Angiotensin-converting-enzyme 2 (ACE2) to selectively capture and rapidly detect SARS-CoV-2 in sewage. This method offers a fast detection speed and an accurate result within 5 minutes. Through such rapid and effective detection, the development of the epidemic can be controlled in a timely manner.

Furthermore, since its unique properties of specifically identifying target molecules, short detection time, simple pretreatment, and the non-contact measurement procedure, Raman spectroscopy has been used in biosensors to detect viruses. More importantly, the portable Raman spectrometer is easy to apply *in situ* and in real time. However, owing to the insufficiency of clinical studies, it is difficult for Raman spectroscopy technology to be practically applied in mass screening for COVID-19. The chemical composition of throat swabs and other real clinical specimens were complex and uneven, which would significantly affect RS detection of SARS-CoV-2. In fact, it is still a challenge for RS to recognize biomolecules due to poor compatibility between biomolecules and RS substrates, complexity of physiological environment, differences between individuals, randomness of biomolecular orientation, and possible interaction between biomolecules and RS substrates. Labeled RS strategy may be an effective method to overcome the complexity of direct measurement, but it has the disadvantages of typical antibody detection methods.

### FTIR spectroscopy sensors

**FIGURE 2:**
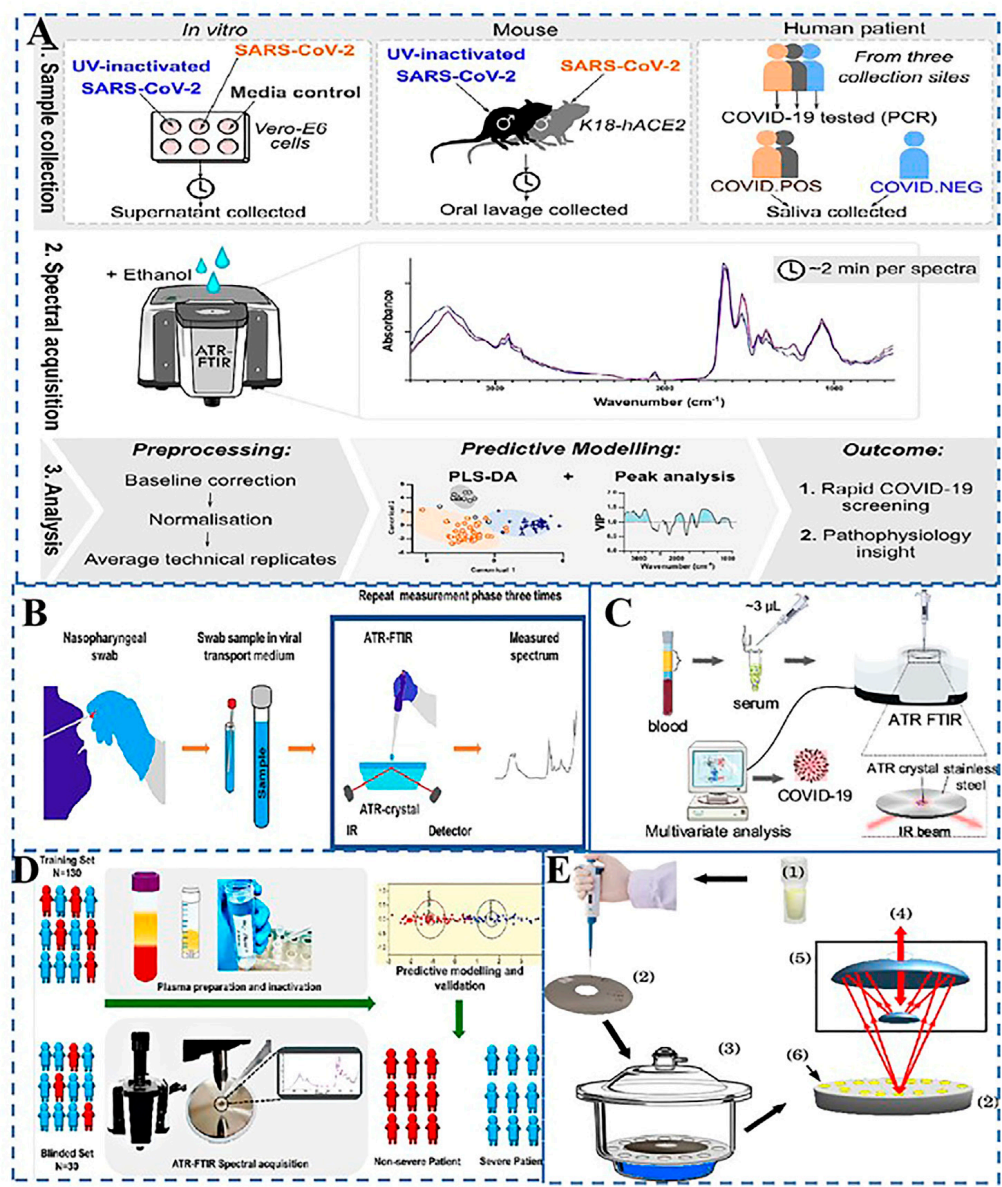
Application of FTIR spectroscopy in virus detection. **(A)** ATR-FTIR spectral changes of culture supernatants, *in vitro* SARS-CoV-2 infection 132 model. Copyright^©^ 2021 Wolters Kluwer Medknow Publications **(B)** Schematic illustration of attenuated total reflection Fourier-transform infrared spectroscopy for detecting COVID-19 from routine nasopharyngeal swab samples Copyright^©^ 2021 Nature Publishing Group **(C)** Diagram of attenuated total reflection Fourier Transform infrared spectroscopy for detection of COVID-19 from blood samples Copyright^©^ 2021 American Chemical Society. **(D)** ATR-FTIR process for rapid blood testing and classification of COVID-19 disease severity in 160 patients with COVID-19 disease. Copyright^©^ 2021 American Chemical Society. **(E)** Scheme for micro-FTIR refectance measurements for human serum. Copyright^©^ 2022 Nature Publishing Group.

Raman spectroscopy irradiates a sample with a monochromatic laser beam, while Fourier transform infrared spectroscopy (FTIR) is based on measuring the absorption of multichromatic infrared light to provide structural and chemical information (lipids, proteins, carbohydrates, and nucleic acids) in biological samples such as tissues, cells, and biological specimens (Erukhimovitch et al., 2004). As far as we concern, numerous reports have discussed the use of FTIR for the detection of viruses, such as herpes viruses, retroviruses and polioviruses (Lee-Montiel et al., 2011). FTIR is a promising, point-of-care detection technique in the detection of COVID-19 by throat swab or saliva detection. According to research by (Kazmer et al., 2021), SARS-CoV-2 infection with good activity induces additional FTIR signal changes on a UV-inactivated SARS-CoV-2 infection, associated with innate immune responses, aggregating proteins and RNA, as shown in Figure 2A. According to Figure 2B, Attenuated Total Reflection (ATR)-FTIR technique is a variant of infrared spectroscopy, which uses evanescent wave generated by total internal reflection of high refractive index crystals to detect sample characteristics in the mid-infrared region. Readily accessible biofluids, such as blood serum, saliva or urine, are considered ideal for clinical implementation as they only require routine methods of collection and minimal sample preparation. Considering the changes in the blood composition of severe COVID-19 patients, Zhang *et al.* figured out that antibodies and phospholipids in serum specimens could behaved in the infrared spectra and showed promising model potential, which indicated ATR-FTIR can be a powerful diagnostic tool for COVID-19 as shown in Figure 2C (Zhang et al., 2021). Encouragement of the scientific community to investigate the potential of this spectrum instrument for spotting changes in biological material, such as those brought on by viruses, was one of the key objectives of (Santos et al., 2021). Barauna *et al.* proposed an ultra-fast reagent free, nondestructive ATR-FTIR method followed by stoichiometric analysis, which was used for the extraction of virus-infected samples (Barauna et al., 2020). The blind sensitivity was 95% and the specificity was 89%. As illustrated in Figure 2D, Banerjee *et al.* demonstrated the potential of ATR-FTIR spectroscopy as a rapid, low-cost triage tool for COVID-19 severity to help manage COVID-19 patients during the pandemic (Banerjee et al., 2021). Even though results from these studies are extremely promising, the number of investigated samples is typically relatively small. Compared to earlier studies, Heino *et al.*,’s analyzed sample set contained 558 positive and 558 negative samples, making it the largest ATR-FTIR study so far to detect the SARS-CoV-2 from the very same samples that were originally collected and processed for the PCR (Heino et al., 2021). To investigate the correlation between antibody response and SARS-CoV-2 infection, serum samples of adult healthcare workers (asymptomatic or mild) infected with COVID-19 and healthy people were detected by Micro-Fourier transform infrared spectroscopy (Micro-FTIR). In Bandeira’s study, Micro-FTIR was used to study serum samples from healthy and COVID-19 (mild or minimally synthetic) individuals (Bandeira, 2022). And the specificity was 87.5% and the true positive detection rate was 100% as shown in Figure 2E.

In short, spectroscopy has been widely used to classify normal and pathological populations using different cell types, tissues, or biological fluids. Analysis of samples with infrared (IR) spectroscopy can produce “spectral fingerprints,” thus facilitating the differentiation of different populations and the identification of potential biomarkers. Biofluid-based ATR-FTIR spectroscopy has been used to diagnose, screen, or monitor the progression/regression of various diseases and the spectroscopy technique is rapid, cost-effective, and non-destructive, which makes it a perfect candidate for translation into the clinic or even as an aid to a more mature approach. However, the measurement results of infrared detection technology are affected by instrument parameters and environmental parameters, so there may be a large error.

### Other optical spectroscopy

In addition to the Raman spectrum and infrared spectrum mentioned above, the other several methods of spectroscopy such as fluorescence spectrum and ultraviolet spectrum have been widely used in various detection methods. Ultraviolet spectroscopy mainly determines the composition of a substance by the position of the absorption peak. A study by Minamikawa *et al.* (2021) used samples of SARS-CoV-2 contained in several different media. The results showed that except PBS without fetal bovine serum, the absorbance peak of all the other media was 280 nm, indicating that the UV absorption of virus was mainly protein. However, UV light has a good effect on virus inactivation, so it cannot be used as an important means of virus detection.

Fluorescence spectroscopy uses the principle that fluorophores in the sample absorb shorter wavelength light and emit longer wavelength light to diagnose the sample. A recent study used fluorescence to detect SARS-CoV-2 antigens or viral particles. This study demonstrated that the ability of Rhodamine 6G (Rh-6G) dye-coupled DNA aptamers attached to gold nanostars (GNS) to rapidly diagnose specific SARS-CoV-2 spinoprotein recombinant antigen or SARS-CoV-2 spinoprotein pseudobaculovirus within 10 min (Pramanik et al., 2021a). To sum up, although these spectral detection methods can provide theoretical means of detection, there is still a long way to go in the field of application.

Although these vibration spectra have different detection methods and required different materials, they all have certain research progress and deficiencies in virus detection. Table 1 summarized the limit of detection, test time, advantages and disadvantages of these four spectroscopic methods in detecting novel coronavirus.

**TABLE 1:** Summary of different detection methods.

**Method**	**Target materials**	**Accuracy of detection**	**Detection time** (min)	**Advantage**	**Disadvantage**	**Ref**
SERS	SARS-CoV-2	300 nM		1. High sensitivity2. Fast detection3. Trace analysis	Additional chemical modifications such as molecular labeling or antibody functionalization are required	Payne et al. (2021)
SERS	COVID-19 viral antigen or virus	Antigen 4 pg/mL Viral particles18 particles/mL	5	1. Fast detection2. Simple operation	The quantity of samples required is large	Pramanik et al. (2021a)
FTIR	SARS-CoV-2		2	1. No damage to the sample 2. Identification structure 3. Check the virus status	The peak value of virus infection in different stages is different, and quantitative detection is difficult	Barauna et al. (2020)
FTIR	SARS-CoV-2			1. Fast detection2. Portable	The results are inaccurate	Wood et al. (2021)
UV	SARS-CoV-2			1. Easy to operate2. Wide application	Ultraviolet light can have a damaging effect on viruses	Minamikawa et al. (2021)
Fluorescence	SARS-CoV-2	Antigen 130 fg/mL Viral particles 8 particles/mL	10	1. Qualitative detection 2. High sensitivity3. Short detection time	Fluorescence annihilation may be caused by interference from other ions	Pramanik et al. (2021b)

### Summary and future perspectives

This article briefly reviewed recent developments of vibrational spectroscopy techniques on the detection of COVID-19. In curbing the disease, early diagnosis is crucial for further prevention and treatment because RNA virus naturally evolve into mutations, so it is unknown whether the coronavirus is the same as its original emergence. It is urgent to come up with a diagnostic method with good efficacy since the alteration of virus make it harder to monitor and find cures. The monitoring of disease progression along chemical pathways and the detection of any potential alterations in the chemical makeup of viruses will be made possible by the use of RS and IR in conjunction with artificial intelligence and machine learning. In addition, it should be able to detect multiple concentrations of the virus as it is able to accurately determine by spectroscopy the changes in chemical structure from saliva, urine, blood or serum of infection. The goal was to create an assay for COVID-19 sample analysis utilizing infrared and Raman spectroscopy, and to compare spectral results with conventional PCR techniques. To sum up, Vibration spectrum can help to understand the infection process, not only realizing fast and accurate detection, but also monitoring the spread of the disease, which may lead to understand its mutation and COVID-19 drugs to develop a diagnostic platform.

In the future, there still exist some problems that need to be solved: 1) The spectroscopic detection of viruses has not yet been used on a large scale in real life. 2) The detection process is fast, but the result feedback is slow. 3) False positives still occur. It is recommended that develop some spectroscopic test paper or kit, can achieve large-scale use. Meanwhile, by combining the test results with mobile software, the test results can be quickly and accurately fed back to the database so as to facilitate data collection and epidemic prevention and control. For the existence of false positive problems, it is necessary to develop good sampling standards to minimize the contamination of other substances.

## References

[B1] Baker M. J. Byrne H. J. Chalmers J. Gardner P. Goodacre R. Henderson A. (2018). Clinical applications of infrared and Raman spectroscopy: State of play and future challenges. Analyst 143 (8), 1735–1757. Available at:. 10.1039/C7AN01871A 29504623

[B2] Bandeira C. C. S. (2022). Micro-Fourier-transform infrared reflectance spectroscopy as tool for probing IgG glycosylation in COVID-19 patients. United Kingdom: Scientific Reports, 13. 10.1038/s41598-022-08156-6 PMC8914452 35277543

[B3] Banerjee A. Gokhale A. Bankar R. Palanivel V. Salkar A. Robinson H. (2021). Rapid classification of COVID-19 severity by ATR-FTIR spectroscopy of plasma samples. Anal. Chem. 93 (30), 10391–10396. Available at:. 10.1021/acs.analchem.1c00596 34279898

[B4] Barauna V. G. Singh M. N. Barbosa L. L. Marcarini W. D. Vassallo P. F. Mill J. G. (2020). Ultra-rapid on-site detection of SARS-CoV-2 infection using simple ATR-FTIR spectroscopy and analysis algorithm: High sensitivity and specificity. Prepr. Public Glob. Health 93, 2950. Available at:. 10.1101/2020.11.02.20223560 33481583

[B5] Bergamaschi G. Borrelli de Andreis F. Aronico N. Lenti M. V. Barteselli C. Merli S. (2021). Anemia in patients with covid-19: Pathogenesis and clinical significance. Clin. Exp. Med. 21 (2), 239–246. Available at:. 10.1007/s10238-020-00679-4 33417082 PMC7790728

[B6] Carlomagno C. Bertazioli D. Gualerzi A. Picciolini S. Banfi P. I. Lax A. (2021). COVID-19 salivary Raman fingerprint: Innovative approach for the detection of current and past SARS-CoV-2 infections. Sci. Rep. 11 (1), 4943. Available at:. 10.1038/s41598-021-84565-3 33654146 PMC7925543

[B7] Cha H. Kim H. Joung Y. Kang H. Moon J. Jang H. (2022). Surface-enhanced Raman scattering-based immunoassay for severe acute respiratory syndrome coronavirus 2. Biosens. Bioelectron. 202, 114008. 10.1016/j.bios.2022.114008 35086030 PMC8770391

[B8] Corman V. M. Landt O. Kaiser M. Molenkamp R. Meijer A. Chu D. K. (2020). Detection of 2019 novel coronavirus (2019-nCoV) by real-time RT-PCR. Eurosurveillance 25 (3), 2000045. Available at:. 10.2807/1560-7917.ES.2020.25.3.2000045 31992387 PMC6988269

[B9] Ember K. Daoust F. Mahfoud M. Dallaire F. Ahmad E. Z. Tran T. (2022). Saliva-based detection of COVID-19 infection in a real-world setting using reagent-free Raman spectroscopy and machine learning. J. Biomed. Opt. 27 (02), 025002. Available at:. 10.1117/1.JBO.27.2.025002 35142113 PMC8825664

[B10] Erukhimovitch V. Talyshinsky M. Souprun Y. Huleihel M. (2004). “FTIR microscopy detection of cells infected with viruses,” in DNA Viruses. Editor Lieberman P. M. (New Jersey: Humana Press) 292, 161–172. 10.1385/1-59259-848-X:161 15507707

[B11] Eskandari V. Sahbafar H. Zeinalizad L. Hadi A. (2022) ‘A review of applications of surface-enhanced Raman spectroscopy laser for detection of biomaterials and a quick glance into its advances for COVID-19 investigations’, ISSS J. Micro Smart Syst. 11, 363, 382. [Preprint]. Available at:. 10.1007/s41683-022-00103-x 35540110 PMC9070975

[B12] Fikiet M. A. Khandasammy S. R. Mistek E. Ahmed Y. Halámková L. Bueno J. (2018). Surface enhanced Raman spectroscopy: A review of recent applications in forensic science. Spectrochimica Acta Part A Mol. Biomol. Spectrosc. 197, 255–260. 10.1016/j.saa.2018.02.046 29496406

[B13] Gao Y. Xiaoxiong W. Rong S. Xiaoming Z. Xiaofei Z. Zhen L. (2023).Promising mass‐productive 4‐inch commercial SERS sensor with particle in micro‐nano porous Ag/Si/Ag structure using in auxiliary diagnosis of early lung cancer. Small, 1, 2207324. Available at:. 10.1002/smll.202207324 36932935

[B14] Goulart A. C. C. Silveira L. Carvalho H. C. Dorta C. B. Pacheco M. T. T. Zângaro R. A. (2022). Diagnosing COVID-19 in human serum using Raman spectroscopy. Lasers Med. Sci. 37 (4), 2217–2226. Available at:. 10.1007/s10103-021-03488-7 35028768 PMC8758209

[B15] Hamilton G. S. (2021). Aerosol‐generating procedures in the COVID era. Respirology 26 (5), 416–418. Available at:. 10.1111/resp.14031 33660369 PMC8014278

[B16] Harrison J. P. Berry D. (2017). Vibrational spectroscopy for imaging single microbial cells in complex biological samples. Front. Microbiol. 8, 675. Available at:. 10.3389/fmicb.2017.00675 28450860 PMC5390015

[B17] Heino H. Rieppo L. Männistö T. Sillanpää M. J. Mäntynen V. Saarakkala S. (2021). Diagnostic performance of attenuated total reflection Fourier-transform infrared spectroscopy for detecting COVID-19 from routine nasopharyngeal swab samples. Sci. Rep. 12, 20358. 10.1101/2021.11.29.21266906 PMC9701801 36437268

[B18] Jadhav S. A. Biji P. Panthalingal M. K. Murali Krishna C. Rajkumar S. Joshi D. S. (2021). Development of integrated microfluidic platform coupled with Surface-enhanced Raman Spectroscopy for diagnosis of COVID-19. Med. Hypotheses 146, 110356. Available at:. 10.1016/j.mehy.2020.110356 33342643 PMC7669482

[B19] Kashefi-Kheyrabadi L. Nguyen H. V. Go A. Baek C. Jang N. Lee J. M. (2022). Rapid, multiplexed, and nucleic acid amplification-free detection of SARS-CoV-2 RNA using an electrochemical biosensor. Biosens. Bioelectron. 195, 113649. 10.1016/j.bios.2021.113649 34555637 PMC8447555

[B20] Kazmer S. T. Hartel G. Robinson H. Richards R. S. Yan K. Van Hal S. J. (2021). Pathophysiological response to SARS-CoV-2 infection detected by infrared spectroscopy enables rapid and robust saliva screening for COVID-19. Biomedicines 10 (2), 351. 10.3390/biomedicines10020351 PMC8962262 35203562

[B21] Khan S. Ullah R. Khan A. Ashraf R. Ali H. Bilal M. (2018). Analysis of Hepatitis B virus infection in blood sera using Raman spectroscopy and machine learning. Photodiagnosis Photodyn. Ther. 23, 89–93. Available at:. 10.1016/j.pdpdt.2018.05.010 29787817

[B22] Leal L. B. Nogueira M. Canevari R. Carvalho L. (2018). Vibration spectroscopy and body biofluids: Literature review for clinical applications. Photodiagnosis Photodyn. Ther. 24, 237–244. Available at:. 10.1016/j.pdpdt.2018.09.008 30282049

[B23] Lee-Montiel F. T. Reynolds K. A. Riley M. R. (2011). Detection and quantification of poliovirus infection using FTIR spectroscopy and cell culture. J. Biol. Eng. 5 (1), 16. Available at:. 10.1186/1754-1611-5-16 22142483 PMC3260089

[B24] Liu G. Mu Z. Guo J. Shan K. Shang X. Yu J. (2022). Surface-enhanced Raman scattering as a potential strategy for wearable flexible sensing and point-of-care testing non-invasive medical diagnosis. Front. Chem. 10, 1060322. Available at:. 10.3389/fchem.2022.1060322 36405318 PMC9669362

[B25] Lukose J. Barik A. K. Unnikrishnan V. George S. D. Kartha V. Chidangil S. (2021). Development of a spectroscopic technique that enables the saliva based detection of COVID-19 at safe distances. Results Chem. 3, 100210. Available at:. 10.1016/j.rechem.2021.100210 PMC8500476 34642620

[B26] Mandrile L. Rotunno S. Miozzi L. Vaira A. M. Giovannozzi A. M. Rossi A. M. (2019). Nondestructive Raman spectroscopy as a tool for early detection and discrimination of the infection of tomato plants by two economically important viruses. Anal. Chem. 91 (14), 9025–9031. 10.1021/acs.analchem.9b01323 31265250

[B27] Minamikawa T. Koma T. Suzuki A. Mizuno T. Nagamatsu K. Arimochi H. (2021). Quantitative evaluation of SARS-CoV-2 inactivation using a deep ultraviolet light-emitting diode. Sci. Rep. 11 (1), 5070. Available at:. 10.1038/s41598-021-84592-0 33658595 PMC7930116

[B28] Movasaghi Z. Rehman S. ur Rehman Dr.I. (2008). Fourier transform infrared (FTIR) spectroscopy of biological tissues. Appl. Spectrosc. Rev. 43 (2), 134–179. Available at:. 10.1080/05704920701829043

[B29] Oliver D. (2021)., 19. BMJ, n2013. Available at:. 10.1136/bmj.n2013 David Oliver: Were Nightingale units and fever hospitals ever workable responses to Covid-19? 34407959

[B30] Pagel C. (2021). Schools: A gaping hole in england’s Covid strategy. BMJ 374, n2115. Available at:. 10.1136/bmj.n2115 34470743

[B50] Payne T. D. Klawa S. J. Jian T. Kim S. H. Papanikolas M. J. Freeman R. (2021). Catching COVID: Engineering peptide-modified surface-enhanced Raman Spectroscopy Sensors for SARS-CoV-2. ACS Sens. 6 (9), 3436–3444. 10.1021/acssensors.1c01344 34491043 PMC8442610

[B31] Pence I. Mahadevan-Jansen A. (2016). Clinical instrumentation and applications of Raman spectroscopy. Chem. Soc. Rev. 45 (7), 1958–1979. Available at:. 10.1039/C5CS00581G 26999370 PMC4854574

[B32] Pereira N. L. Ahmad F. Byku M. Cummins N. W. Morris A. A. Owens A. (2021). COVID-19: Understanding inter-individual variability and implications for precision medicine. Mayo Clin. Proc. 96 (2), 446–463. 10.1016/j.mayocp.2020.11.024 33549263 PMC7713605

[B33] Pramanik A. Gao Y. Patibandla S. Mitra D. McCandless M. G. Fassero L. A. (2021a). Aptamer conjugated gold nanostar-based distance-dependent nanoparticle surface energy transfer spectroscopy for ultrasensitive detection and inactivation of corona virus. J. Phys. Chem. Lett. 12 (8), 2166–2171. 10.1021/acs.jpclett.0c03570 33629859 PMC7927280

[B34] Pramanik A. Gao Y. Patibandla S. Mitra D. McCandless M. G. Fassero L. A. (2021b). The rapid diagnosis and effective inhibition of coronavirus using spike antibody attached gold nanoparticles. Nanoscale Adv. 3 (6), 1588–1596. Available at:. 10.1039/D0NA01007C 34381960 PMC8323809

[B35] Qiblawey Y. Tahir A. Chowdhury M. E. H. Khandakar A. Kiranyaz S. Rahman T. (2021). Detection and severity classification of COVID-19 in CT images using deep learning. Diagnostics 11 (5), 893. Available at:. 10.3390/diagnostics11050893 34067937 PMC8155971

[B36] Revin V. Balykova L. Pinyaev S. Syusin I. Radaeva O. Revina N. (2022). Research into morphofunctional characteristics of erythrocytes in COVID-19 patients. Biomedicines 10 (3), 553. Available at:. 10.3390/biomedicines10030553 35327355 PMC8945033

[B37] Robertson J. L. Senger R. S. Talty J. Du P. Sayed-Issa A. Avellar M. L. (2022). Alterations in the molecular composition of COVID-19 patient urine, detected using Raman spectroscopic/computational analysis’, *PLOS ONE* . by B. Bussolati 17 (7), e0270914. Available at:. 10.1371/journal.pone.0270914 PMC9292080 35849572

[B38] Santos M. C. D. Morais C. L. M. Lima K. M. G. (2021). ATR-FTIR spectroscopy for virus identification: A powerful alternative. Biomed. Spectrosc. Imaging 9 (3–4), 103–118. Available at:. 10.3233/BSI-200203

[B39] Talari A. C. S. Movasaghi Z. Rehman S. Rehman I. u. (2015). Raman spectroscopy of biological tissues. Appl. Spectrosc. Rev. 50 (1), 46–111. Available at:. 10.1080/05704928.2014.923902

[B40] Tong D. Chen C. Zhang J. Lv G. Zheng X. Zhang Z. (2019). Application of Raman spectroscopy in the detection of Hepatitis B virus infection. Photodiagnosis Photodyn. Ther. 28, 248–252. Available at:. 10.1016/j.pdpdt.2019.08.006 31425766

[B41] Wang D. Xu G. Zhang X. Gong H. Jiang L. Sun G. (2022). Dual-functional ultrathin wearable 3D particle-in-cavity SF-AAO-Au SERS sensors for effective sweat glucose and lab-on-glove pesticide detection. Sensors Actuators B Chem. 359, 131512. 10.1016/j.snb.2022.131512

[B42] WHO Coronavirus (2022). COVID-19) dashboard . Available at: https://covid19.who.int (Accessed: August 29, 2022).

[B43] Wiersinga W. J. Rhodes A. Cheng A. C. Peacock S. J. Prescott H. C. (2020). Pathophysiology, transmission, diagnosis, and treatment of coronavirus disease 2019 (COVID-19): A review. JAMA 324 (8), 782. Available at:. 10.1001/jama.2020.12839 32648899

[B49] Wood B. R. Kochan K. Bedolla D. E. Salazar-Quiroz N. Grimley S. L. Perez-Guaita D. (2021). Infrared based saliva screening test for COVID-19. Angew. Chem. 133 (31), 17239–17244. 10.1002/ange.202104453 PMC8222893 34043272

[B44] Yakoh A. Pimpitak U. Rengpipat S. Hirankarn N. Chailapakul O. Chaiyo S. (2021). Paper-based electrochemical biosensor for diagnosing COVID-19: Detection of SARS-CoV-2 antibodies and antigen. Biosens. Bioelectron. 176, 112912. Available at:. 10.1016/j.bios.2020.112912 33358057 PMC7746088

[B45] Yang Y. Peng Y. Lin C. Long L. Hu J. He J. (2021). Human ACE2-functionalized gold “virus-trap” nanostructures for accurate capture of SARS-CoV-2 and single-virus SERS detection. Nano-Micro Lett. 13 (1), 109. Available at:. 10.1007/s40820-021-00620-8 PMC8042470 33868761

[B46] Yin G. Li L. Lu S. Yin Y. Su Y. Zeng Y. (2021). An efficient primary screening of COVID‐19 by serum Raman spectroscopy. J. Raman Spectrosc. 52 (5), 949–958. Available at:. 10.1002/jrs.6080 33821082 PMC8014023

[B47] Zhang L. Xiao M. Wang Y. Peng S. Chen Y. Zhang D. (2021). Fast screening and primary diagnosis of COVID-19 by ATR–FT-IR. Anal. Chem. 93 (4), 2191–2199. Available at:. 10.1021/acs.analchem.0c04049 33427452

[B48] Zhang X. Young M. A. Lyandres O. Van Duyne R. P. (2005). Rapid detection of an anthrax biomarker by surface-enhanced Raman spectroscopy. J. Am. Chem. Soc. 127 (12), 4484–4489. Available at:. 10.1021/ja043623b 15783231

